# Improvised grey wolf optimizer assisted artificial neural network (IGWO-ANN) predictive models to accurately predict the permeate flux of desalination plants

**DOI:** 10.1016/j.heliyon.2024.e34132

**Published:** 2024-07-05

**Authors:** Rajesh Mahadeva, Mahendra Kumar, Anjali Diwan, Gaurav Manik, Saurav Dixit, Gobind Das, Vinay Gupta, Anuj Sharma

**Affiliations:** a Department of Physics, Khalifa University, Abu Dhabi, 127788, United Arab Emirates; b Department of Instrumentation and Control Engineering, Dr. B R Ambedkar National Institute of Technology, Jalandhar, Punjab, 144011, India; c Department of CE-AI, Marwadi University, Rajkot, Gujarat 360003, India; d Department of Polymer and Process Engineering, Indian Institute of Technology, Roorkee, Uttarakhand, 247667, India; e Division of Research and Development, Lovely Professional University, Phagwara, Punjab, 144401, India; f Jindal Global Business School, O. P. Jindal Global University, Sonipat, Haryana, 131001, India

**Keywords:** Desalination, Artificial intelligence (AI) technologies, Algorithm, Optimization

## Abstract

Effective planning, management, and control of industrial plants and processes have exploded in popularity to enhance global sustainability in recent decades. In this arena, computational predictive models have significantly contributed to plant performance optimization. In this regard, this research proposes an Improvised Grey Wolf Optimizer (IGWO) aided Artificial Neural Network (ANN) predictive model (IGWO-ANN Model-1 to 4) to predict the performance (permeate flux) of desalination plants accurately. For this, the proposed models investigated experimental inputs four: salt concentration & feed flow rate, condenser & evaporator inlet temperatures of the plant. Besides, mean squared error (MSE) and the regression coefficients (R^2^) have been used to assess the models' accuracy. The proposed IGWO-ANN Model-4 shows strong optimization abilities and provides better R^2^ = 99.3 % with minimum errors (0.004) compared to existing Response Surface Methodology (RSM) (R^2^ = 98.5 %, error = 0.100), ANN (R^2^ = 98.8 %, error = 0.060), GWO-ANN (R^2^ = 98.8 % error = 0.008), models. The proposed models are multitasking, multilayers, and multivariable, capable of accurately analyzing the desalination plant's performance, and suitable for other industrial applications. This study yielded a promising outcome and revealed the significant pathways for the researchers to analyze the desalination plant's performance to save time, money, and energy.

## 1 Introduction

### 1.1 Background

Water desalination has emerged as a critical solution for addressing the world's rising water scarcity issues. As freshwater resources deplete owing to causes such as population expansion, climate change, and pollution, the demand for alternate water sources has never been higher. Desalination is a dependable method of transforming saline water, primarily seawater and brackish water, into drinkable water, giving a long-term solution for many water-stressed locations worldwide. Desalination is critical for delivering fresh water in desert regions, small islands, and coastal communities where natural freshwater sources are limited. Sustainable agricultural activities, industrial processes, and municipal water supplies are also necessary. Countries such as Saudi Arabia, the United Arab Emirates, and Israel rely extensively on desalination to meet their water needs. In recent decades, industrial plant planning, development, management, and control have become prominent research areas of researchers toward global sustainability [[1], [2], [3]].

The two basic desalination processes are (1) Thermal Desalination: Multi-Stage Flash (MSF) Distillation, which involves heating seawater and flashing it into steam in numerous stages. The steam is subsequently condensed, yielding fresh water. Multiple Effect Distillation (MED) is similar to MSF, except it uses several vessels (effects) to boil repeatedly and condense seawater. Vapor Compression Distillation (VCD) compresses and condenses vapor using mechanical or thermal energy to produce fresh water. (2) Membrane Desalination: Reverse Osmosis (RO) is the most commonly used desalination technology. It involves driving seawater through semi-permeable membranes that filter out salt and other pollutants. Electrodialysis (ED) and Electrodialysis Reversal (EDR) use electrical potential to transport salt ions through selective membranes, separating them from freshwater [4,5].

Literature suggests that artificial intelligence (AI) technologies are helping to improve plant design to save time, money, and energy [[6], [7], [8], [9]]. Various AI technologies and optimization models, such as artificial neural networks (ANN) [[10], [11], [12], [13], [14], [15]], fuzzy logic (FL) [16,17], grey wolf optimizer (GWO) [18], genetic algorithm (GA) [11,19], particle swarm optimization (PSO) [16,[20], [21], [22]], whale optimization algorithm [23], deep network [24], controller design [25,26], optimization techniques [27], are playing a vital role in the plant's performance. Literature also suggests that the systematic approach and the appropriate selection of the modeling parameters make the model perfect [21]. For this, it is essential to understand the model's characteristics and the required modeling parameters well. Therefore, the researchers are quite interested in designing an optimized model.

### 1.2 Literature review

Various artificial intelligence models have been developed previously in multiple fields. Some of these models are mentioned here in detail to understand the research more closely and give an overview of the subjects under consideration. Li et al. (2021) [18] developed a GWO-based backpropagation neural network (BPNN) model to predict accurately (thermal comfort levels). Models used 100 to 250 grey wolves and 1000 to 2000 iterations and found better outcomes than the GA-ANN and PSO-ANN models. The proposed models have an excellent optimization to predict thermal comfort levels and achieve 1.01 % energy savings. Likewise, Bahiraei et al. (2021) [28] presented eight optimization models for predicting heat transfer rate: Artificial Bee Colony (ABC-ANN), Whale Optimization Algorithm (WOA-ANN), Ant Colony Optimization (ACO-ANN), Harris Hawks Optimizer (HHO-ANN), Ant Lion Optimizer (ALO-ANN), Biogeography Based Optimization (BBO-ANN), GWO-ANN, & Dragonfly Algorithm (DA-ANN). Models have been employed with 25–500 population size, 1000 iterations, 3 hidden layer neurons (*n* = 3), and in their modeling approach, 70 % of the datasets were used for training, while 30 % were used for testing. They found that the 300, 500, 400, 250, 25, 350, 300, and 450 were the most optimum population sizes for the HHO-ANN, GWO-ANN, WOA-ANN, ABC-ANN, ACO-ANN, ALO-ANN, BBO-ANN, and DA-ANN models, respectively.

Similarly, Maroufpoor et al. (2021) [29] developed an ANN-GWO model for estimating the hydrological cycle's reference evapotranspiration. The model has employed 31 provinces with five distinct climate datasets from Iran. They used 15 to 30 population sizes and 200 to 1500 iterations in their modeling and discovered that 30 population sizes and 1500 iterations produced the best outcomes. Finally, they reported that the proposed model was much more accurate than the least square support vector regression (LS-10.13039/100019572SVR) and ANN models with minimum errors. Cui et al. (2022) [30] suggested an {Adaptive Neuro Fuzzy Inference System with an Improved Alpha - Grey Wolf Optimizer (ANFIS-IA-GWO)} for accurate prediction of groundwater level. They used 31 years of monthly datasets (1981–2011) for the modeling. The datasets were divided into 70 percent training (Jan. 1981 to Aug. 2002) and 30 percent testing (Sept. 2002 to Dec. 2011). The simulation findings suggest that the proposed models work well with large datasets and perform better than ANN, ANN-PSO, ANN-IA-GWO, ANFIS, and ANFIS-PSO models. Recently, Alardhi et al. (2024) [31] developed ANN and RSM models to predict the conductivity (μs/cm) of the thermal power stations west water. The ANN architecture used two hidden layers, such as 4-20-30-1, in this research. In addition, the LM-BP training algorithm has been used for the ANN optimization.

### 1.3 Motivation

According to the literature, the models' accuracy depends on the choice of modeling parameters & algorithms. Following the literature studied earlier, nature-inspired algorithms (e.g., PSO, GA, GWO, ACO, etc.) offer strong search capabilities for achieving global optima. Furthermore, these algorithms can adapt to objective functions. They can also be used for both linear and nonlinear systems. Due to their exceptionality and capacity to identify the ideal weights and biases in global optima, GWO algorithms are given special attention in this work. As a result, this research developed an Improvised GWO (IGWO) algorithm to attain global optima for ANN with minimal errors. This study examined datasets from reverse osmosis (RO) desalination plants and validated the findings using existing models.

### 1.4 Objectives and contributions

The main objective of this study is to examine how artificial intelligence (AI) technologies are used in water desalination plants. Many academics have studied this field and developed numerous models for enhancing plant outcomes and performance. However, to the best of our knowledge, this IGWO-ANN technique is being proposed and applied for the first time in an RO desalination plant (based on a literature study). Nonetheless, based on a literature study, this IGWO-ANN technique is being suggested and used for the first time in an RO desalination plant. The secondary focus of this research is to enhance the fields of water desalination for a more sustainable future. As WHO and UNICEF reported [[32], [33], [34]], four billion people are affected by water shortage. Therefore, this research included desalination plant datasets to encourage this field. The final goal of this study is to use updated algorithms to determine the ideal weights and biases for ANN to achieve the best outcomes.

The significant contributions of this study are the following: (1) It presents a novel hybrid IGWO-ANN model for accurately predicting desalination plant performance. (2) It uses an improvised GWO approach to find the best biases and weights for the ANN model, considerably improving the desalination plant's performance. (3) The proposed models predict and optimize performance faster than existing models (ANN, RSM [35], and GWO-ANN [36]).

## 2 Materials and methods

### 2.1 Data description

This study's modeling analysis used earlier studies' water desalination plant datasets (Gil et al., 2018) [35]. Four input variables were considered: feed flow rate and salt content; intake temperatures for the evaporator and condenser; and permeate flux, which was determined as a plant output and shown in (Table 1). The Fraunhofer Institute designed this plant module for Solar Energy Systems, and it was sold by Solar Spring (Freiburg, Germany). It involved a commercial membrane from W. L. Gore Associates with a polytetrafluoroethylene (PTFE) layer called a Permeate Gap Membrane Distillation (PGMD) system. Furthermore, the obtained datasets were organized suitably according to the modeling requirements and divided into training (75 %), validation (20 %), and testing (05 %). Literature suggests [7,10,16] various combinations of the dataset divisions, but this research followed Gil et al.'s dataset division to validate the proposed IGWO-ANN model. Besides, the major changes (adding new modeling parameters and strategies) have been highlighted in the following sections (results and discussion sections and the methodology sections) with appropriate justifications.

**Table 1:** Input-output parameters are involved in this modeling investigation of the plants [35].

A. Input parameters	Value/Range
1. Feed flow rate (*F*)	400 L/h – 600 L/h
2. Evaporator inlet temp. (*T*_evap_)	60 °C – 80 °C
3. Salt concentration (*S*)	35 g/L – 140 g/L
4. Condenser inlet temp. (*T*_cond_)	20 °C – 30 °C
B. Output parameter	
1. Permeate flux (*P*_flux_)	0.118 L/h⋅m^2^ – 2.656L/h⋅m^2^

### 2.2 Methodology

#### 2.2.1 Artificial neural network (ANN)

An artificial neural network is a computational model based on the biological neural networks of the human brain. ANNs are intended to identify patterns, learn from data, and make judgments or predictions. They are widely utilized in various applications, including image and speech recognition, medical diagnosis, financial forecasting, and, more recently, optimizing industrial processes such as desalination. An artificial neural network is made up of layers of interconnected nodes or neurons. Each neuron processes input information and sends the results to neurons in the following layer [1,2,6,7].

**Fig. 1:**
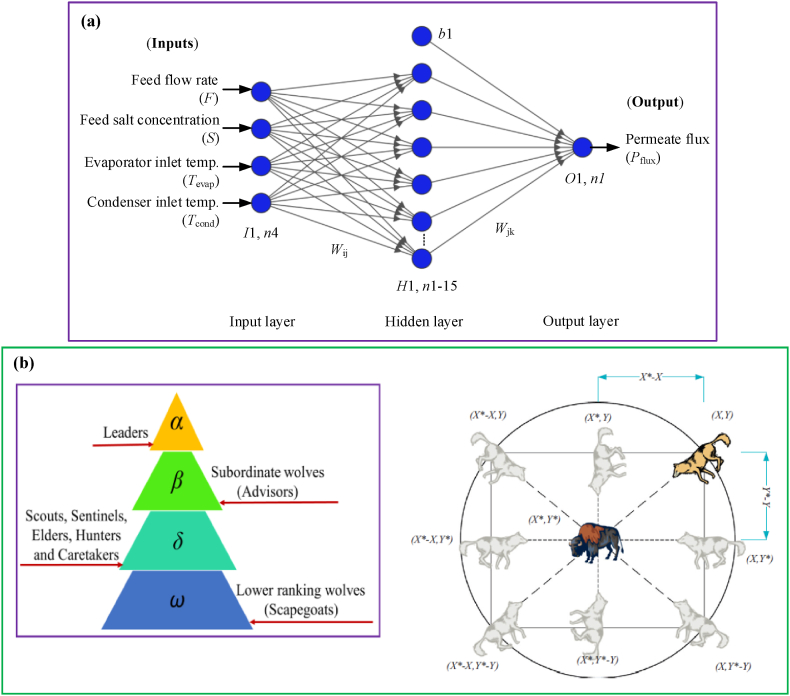
(a). Proposed IGWO-ANN structural design {(*I*1, *n*4): (*H*1, *n*1–15): (*O*1, *n*1)}. Where, (*I*1, *n*4) ≈ one input layer with four neurons; (*H*1, *n*1–15) ≈ one hidden layer with varying from 1 to 15 hidden layer neurons; (*O*1, *n*1) ≈ one output with one neuron; *b*1 ≈ one bias; *W*_ij_ ≈ weights (between input & hidden layers); and *W*_jk_ ≈ weights (between hidden & output layers). **(b).** Illustration of the leadership hierarchy of the grey wolves and the hunting behavior (in 2D position vector). Photo: Courtesy Mirjalili et al., 2014 [38].

The ANN is an intelligent technique/tool based on the function of a human's nervous system. It was first developed by Rosenblatt (1958) [37] to calculate the computational method. It is now widely employed in practically all technical and medical disciplines due to its ease of processing and precise prediction capabilities. In general, the multilayer perceptron neural network (MLP) architecture has been employed with input, output, and hidden layers in the center coupled with neurons, weights, and biases, as displayed in Fig. 1 (a). The neurons receive inputs from the first input layer, while the hidden layers are responsible for learning complex features, and the weights and biases (associated with the input layer) adjust the inputs based on their impact on the output. Finally, it establishes the connections between the input and output data. The BP algorithm is generally used in the conventional ANN model to generate weights. However, many new nature-inspired algorithms have obtained the best weights in recent years. To summarize, ANN is a suitable and powerful tool for optimizing various engineering and medical problems, capable of producing precise results. This research presented classic architecture based on the literature inspiration {(*I*1, *n*4): (*H*1, *n*1–15): (*O*1, *n*1)} as shown in Fig. 1 (a). In this structure, (*I*1, *n*4) is presented one input layer associated with 4 neurons, (*H*1, *n*1–15) is presented one hidden layer associated with varying 1 to 15 hidden layer neurons and (*O*1, *n*1) is presented one output layer associated with one neuron.

#### 2.2.2 Improvised grey wolf optimizer (IGWO)

Mirjalili et al. (2014) [38] developed the initial GWO algorithm, which is influenced by grey wolf hierarchy leadership and hunting behavior. It consists of three steps: (i) looking for prey, (ii) circling, and (iii) attacking. The grey wolves are separated into four groups: (1) alpha (*α*), (2) beta (*β*), (3) delta (*δ*), and (4) omega (*ω*). Fig. 1 (b) depicts the grey wolves' entire hunting behavior (in 2D position vector) and leadership hierarchy. As exposed in Fig. 1 (b), alpha wolves hold the largest social responsibility; they make all decisions, and other wolves must obey their directions. In decision-making, beta wolves assist the alpha. The omega wolves are the bottom-level wolves. These wolves are the last to take food and are obedient to other wolves. The wolves are known as delta, other than the alpha, beta, and omega groups. Alpha and beta wolves lead the delta wolves, who are responsible for scouting, protecting, hunting, and caring for the group.

The IGWO algorithm's mathematical equations are as follows:

As stated before in this section, the grey wolf's hunting behavior consists of three steps: (i) looking for prey, (ii) circling, and (iii) attacking. Based on these steps, the mathematical equation can be defined as follows.

Step (i) Looking for prey stage: In this first stage, grey wolves search and track their prey for hunting.

Step (ii) Stage of circling: Grey wolves circle their prey to hunt after completing the first stage. The following mathematical equations describe their circling behavior:

(1) $$\vec{D}=\left|\vec{C}.{\vec{X}}_{p}\left(i\right)-\vec{X}\left(i\right)\right|;\;\vec{X}\left(i+1\right)={\vec{X}}_{p}\left(i\right)-\vec{A}.\vec{D}$$

where,

${\vec{X}}_{p}$ = prey's position (vectors); i = present iteration; $\vec{X}$ = grey wolves' position (vectors); $\vec{A}$ and $\vec{C}$ = coefficient (vectors weighted); ${\vec{r}}_{1}$ and ${\vec{r}}_{2}$ = random vectors in (0,1); $\vec{A}=2\vec{a}.{\vec{r}}_{1}-\vec{a}$ and $\vec{C}=2.{\vec{r}}_{2}$; $\vec{a}=2\left(1-\frac{i^{2.5}}{i_{m}^{2.5}}\right)$; and $i_{m}$ = max. iterations. (*First modification has been done in the $\vec{a}$ *function; details are discussed in the subsection “Modifications in the initial GWO algorithm are as follows:*“).

Step (iii) Attacking grey wolves stage: In the first and second stages, grey wolves trace and encircle the prey, and in the third stage, they are ready to attack. As previously stated, alpha wolves lead the groups, while beta, delta, and omega wolves are the next-to-last alternatives regarding their importance. From a mathematical perspective, alpha, beta, and delta have potential evidence regarding the prey's location (in the search space). Consequently, based on their ranking inside the group, the three options for the top search agents. As a result, equation (1) now describes updates:

(2) $${\vec{D}}_{\alpha }=\left|{\vec{C}}_{1}.{\vec{X}}_{\alpha }\left(i\right)-\vec{X}\left(i\right)\right|;\;{\vec{D}}_{\beta }=\left|{\vec{C}}_{2}.{\vec{X}}_{\beta }\left(i\right)-\vec{X}\left(i\right)\right|;\;{\vec{D}}_{\delta }=\left|{\vec{C}}_{3}.{\vec{X}}_{\delta }\left(i\right)-\vec{X}\left(i\right)\right|$$

(3) $${\vec{X}}_{1}={\vec{X}}_{\alpha }-{\vec{A}}_{1}.{\vec{D}}_{\alpha }\;;\;{\vec{X}}_{2}={\vec{X}}_{\beta }-{\vec{A}}_{2}.{\vec{D}}_{\beta };\;{\vec{X}}_{3}={\vec{X}}_{\delta }-{\vec{A}}_{3}.{\vec{D}}_{\delta }$$

The following is the final, improved position vector for hunting grey wolves:

(4) $$\vec{X}\left(i+1\right)=\frac{3{\vec{X}}_{1}+2{\vec{X}}_{2}+{\vec{X}}_{3}}{6}$$

where, ${\vec{X}}_{1}$, ${\vec{X}}_{2}$, and ${\vec{X}}_{3}$ are best solutions for *α*, *β,* and *δ* wolves at $i^{th}$ iteration and ${\vec{A}}_{1}$, ${\vec{A}}_{2}$, ${\vec{A}}_{3}$, ${\vec{C}}_{1}$, ${\vec{C}}_{2}$ and ${\vec{C}}_{3}$ are the coefficient vectors. (**Second modification has been done in the $\vec{X}\left(i+1\right)$ *function; details discussed in the subsection “Modifications in the initial GWO algorithm is as follows:“*).

Modifications in the initial GWO algorithm are as follows:

There are two modifications have done in the initial GWO algorithm as follows.

1. Literature noticed that, in the initial GWO algorithm [38], 50:50 percent of the maximum iterations are allocated to the exploration phase (when $\left|\vec{A}\right|\geq 1$) and the exploitation phase (when $\left|\vec{A}\right|\leq 1$). In this regard, the literature suggests [39] (i) Excessive exploration may lessen the risk of becoming stuck (in the local optimum solution), but it also introduces a high level of variability and randomness. As a result, it is possible that the finest selection may be missed. (ii) Excessive exploitation is associated with less randomness, and it may be possible that it will not provide the global best answer. Thus, with the literature's motivation, this research improvised the percentage balance of the exploration and exploitation phases in the IGWO algorithm. As a result, it found that the IGWO algorithm performs superior outcomes than the initial GWO algorithm. This research allocated more percentages of the maximum iterations to the exploration phase than the exploitation phase in the IGWO algorithm. This research considered the linear decreasing function $\vec{a}=2\left(1-\frac{i^{2.5}}{i_{m}^{2.5}}\right)$ in place of $\vec{a}=2\left(1-\frac{i}{i_{m}}\right)$.
2. Literature also noted that all grey wolf groups (alpha, beta, delta, and omega) have the same weightage in the original GWO algorithm. However, alpha is known to be more powerful than others in social and decision-making contexts. Therefore, in the IGWO algorithm, this research has given alpha wolves more weight than others. The grey wolves' weightage is decreasing as per their priority in their group. As a result, the final improved position vector of hunting grey wolves is shown as: $\vec{X}\left(i+1\right)=\frac{3{\vec{X}}_{1}+2{\vec{X}}_{2}+{\vec{X}}_{3}}{6}$ in place of $\vec{X}\left(i+1\right)=\frac{{\vec{X}}_{1}+{\vec{X}}_{2}+{\vec{X}}_{3}}{3}$.

By considering both modifications to the initial GWO algorithm, the IGWO algorithm performs better than the initial GWO algorithm. Therefore, this paper prefers an IGWO algorithm to estimate the performance of the desalination plant.

#### 2.2.3 Proposed IGWO -ANN models

**Fig. 2:**
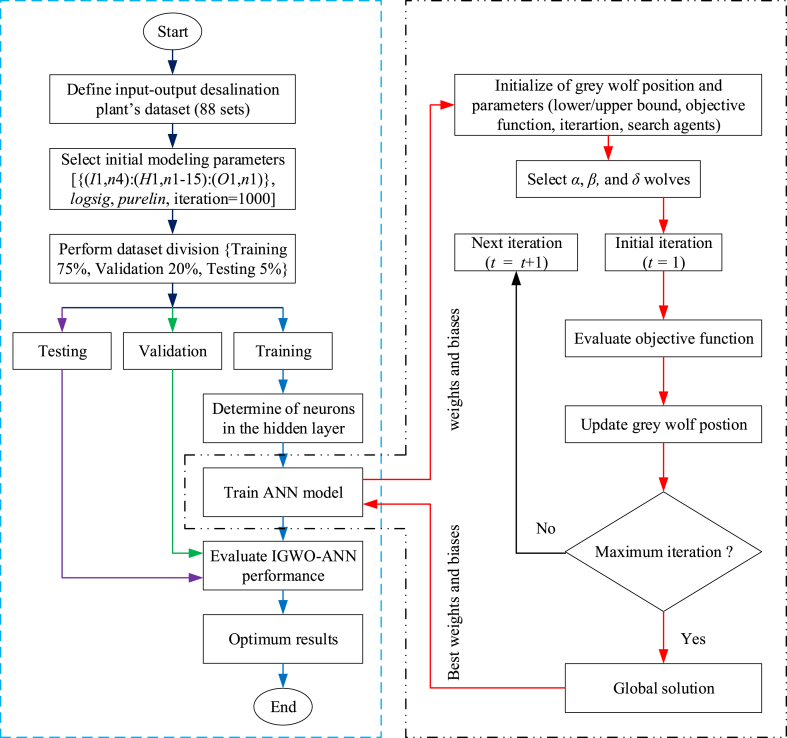
The proposed IGWO-ANN model flowchart.

The proposed IGWO-ANN models are the computational analytical tools that combine the ANN and the IGWO algorithm. As mentioned before, the network in the fundamental ANN design is trained using a BP algorithm. By modifying each neuron's weights and bias values, this approach seeks to reduce the variation between the simulated and observed values. According to the literature, the BP algorithm does not always achieve the desired results. As a result, researchers have discovered several alternative solutions that support ANN architecture. In this context, many nature-inspired algorithms developed by researchers (e.g., PSO, GA, GWO, WOA, etc.) play a critical role in ANN modeling. The IGWO algorithm is used in this investigation because of its effective characteristics and capacity to provide optimal results. A flowchart of the proposed IGWO-ANN model (Fig. 2) has been illustrated.

To start the investigation, gather the datasets for the input-output desalination plants (88 sets total). After that, arrange them properly and select the optimal modeling parameters, which previous studies have determined. In addition, the dataset has been divided into three sections: testing 5 %, training 70 %, and validation 20 %, in line with studies by Gil et al. (2018) to validate the proposed model. The suggested model is now prepared for training. As previously explained in detail, the optimal weights and biases are provided in the subsequent phase by the IGWO algorithm. Additionally, the ANN model was trained using these ideal data. Lastly, assess the performance of the IGWO-ANN and note the best outcomes.

The mean squared error (MSE) is used in this study to assess the fitness function as follows [16,21,40]:

(5) $$\text{MSE}=\min\frac{1}{2N}\sum_{p=1}^{N}\sum_{k=1}^{M}{\left(y_{k}^{p}-{\hat{y}}_{k}^{p}\right)}^{2}$$

where, $y_{k}^{p}$ = presents the actual experimental output; *N* = number of patterns; ${\hat{y}}_{k}^{p}$ = presents the predicted output, *M* = number of output neurons. Literature suggests a minimum MSE value is better & recommends that the model is more accurate. The proposed models were implemented in MATLAB version 2022a (Neural Network toolbox).

## 3 Results and discussion

The findings of the proposed modeling are summarized in this section. ‘Optimization,' ' best performance models,' and ‘comparison with existing models' are the three subsections of this section. Each subsection, one by one, has been adequately described in the following section with fruitful findings. The MSE has evaluated the modeling performances (refer to equation (5)) and regression coefficients (R^2^, refer to equation (6)) to assess prediction accuracy. The mathematical equation for R^2^ is presented as follows [16,20]:

(6) $$R^{2}=1-\frac{\sum_{i=1}^{N}{\left(R_{i}-{\bar{P}}_{a}\right)}^{2}}{\sum_{i=1}^{N}{\left(P_{i}-{\bar{P}}_{a}\right)}^{2}}$$

where, $R_{i}$ = real experimental data, N = number of samples, $P_{i}$ = predicted data, and ${\bar{P}}_{a}$ = average value (of predicted data).

### 3.1 Optimization

There are two key steps in optimizing the proposed models. First, proper model development: as indicated previously in the methodology section, this research chose the optimal modeling parameters suggested by the literature. Second, use the hit-and-error method: a systematic hit-and-error approach makes models perfect. For this, research has selected two modeling parameters {(*n* and search agents (*SA*)} that have more influence on the modeling. According to the literature, they are most responsible for the modeling's success. As a result, a good number of *n* and *SA* are required for model optimization.

#### 3.1.1 Selection of the best hidden layer neurons (n)

**Fig. 3:**
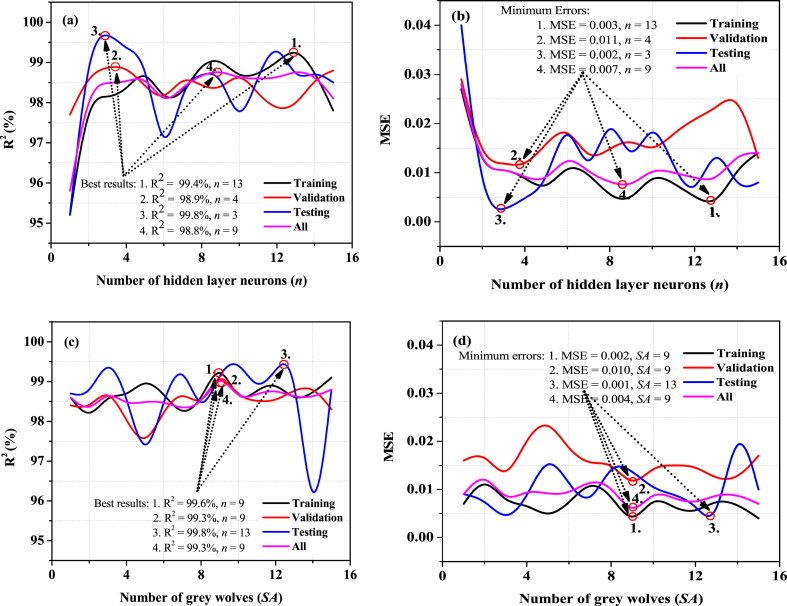
The effect of the number of hidden layer neurons (*n*) and the number of grey wolves (*SA*) in the proposed model's distinct stages: **(a,c)** Best performance in R^2^ (%), **(b,d)** Minimum errors (in MSE).

This research systematically modifies the hidden layer neurons (*n* = 1 to 15) to determine the proposed model's optimal parameters, as shown in (Table 2). This research discovered a variety of outcomes for each stage (training, validation, testing, and all). This research found that *n* = 13 had the finest result with the least error (R^2^ = 99.4 %, MSE = 0.003) for the training stage; *n* = 4 had the finest result with the least amount of error (R^2^ = 98.9 %, MSE = 0.011) during the validation stage; *n* = 3 had the greatest performance with the least amount of error (R^2^ = 99.8 %, MSE = 0.002) during the testing stage; and *n* = 9 had the best performance with the least amount of error (R^2^ = 98.8 %, MSE = 0.007) for all stage. This research has observed that the testing results were the best-of-the-best, with a near-zero error rate compared to others. Finally, Fig. 3 (a,b) shows the effect of the *n* on the proposed model's individual stages (training, validation, testing, and all).

**Table 2:**
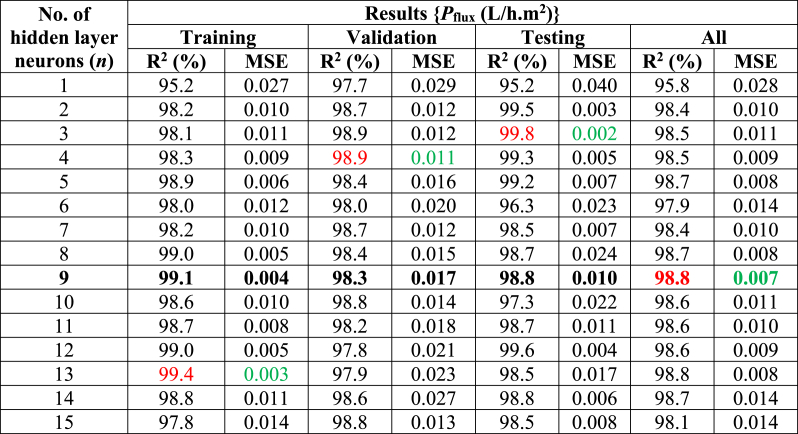
Selection of the proposed model's hidden layer neurons (*n*) in different stages. Minimum MSE (highlighted green color), Maximum R^2^ (%) (highlighted red color), and Best performance (highlighted in red & green color with bold).

#### 3.1.2 Selection of the best number of grey wolves

In the second choice, the recommended modeling contains the optimum value of gray wolves/search agents (*SA*). As shown in (Table 3), this research adjusts the (*SA* = 1 to 15) to select the optimal parameters for the proposed model. This research has observed that the effect of grey wolves (*SA*) differs for training, validation, testing, and all stages. The *SA* = 9 had the greatest performance with minimal error for the training stage (R^2^ = 99.6 %, MSE = 0.002), validation stage (R^2^ = 99.3 %, MSE = 0.010), and all stages (R^2^ = 99.3 %, MSE = 0.004), while the *SA* = 13 had best with minimum error for the testing stage (R^2^ = 98.9 %, MSE = 0.011). The testing stage yielded the best results, coming close to zero error for the proposed model. Finally, Fig. 3 (c,d) shows the effect of the SA on the proposed model's distinct stages (training, validation, testing, and all).

**Table 3:**
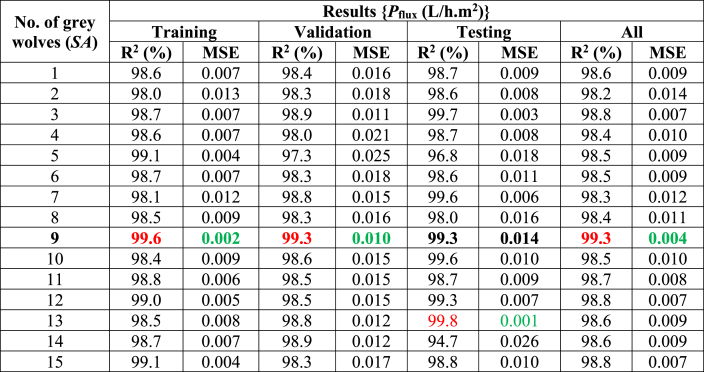
Selection of the proposed model's grey wolves (SA) in different stages: Minimum MSE (highlighted green color), Maximum R^2^ (%) (highlighted red color), and Best performance (highlighted in red & green color with bold).

For both cases (Optimal value of *n* and *SA*), this research found that the testing stage's error is almost close to zero. It demonstrates the best modeling result for the modeling expert and the researchers. In addition, the best search agents recorded 9, and the best hidden layer neurons also recorded 9, for the proposed modeling of the overall stages.

### 3.2 Best performance models

**Fig. 4:**
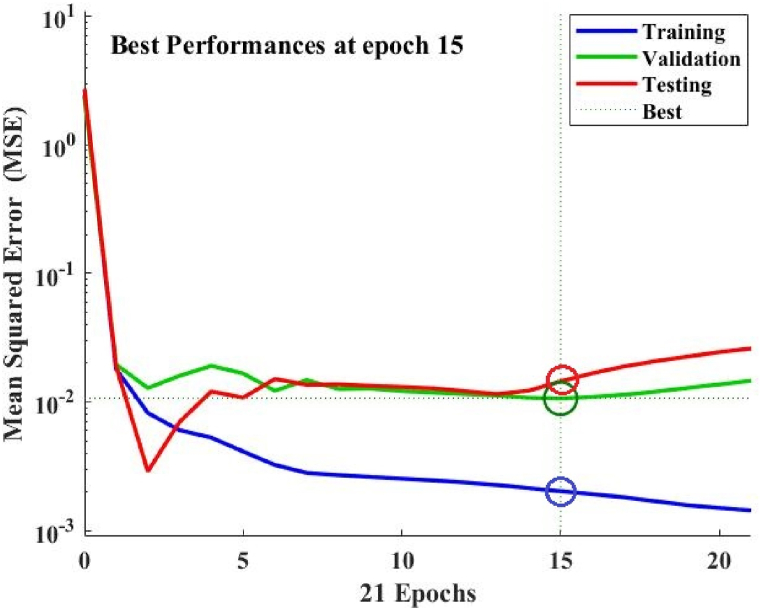
Best results of the proposed model IGWO-ANN Model‒4 at epoch 15.

**Fig. 5:**
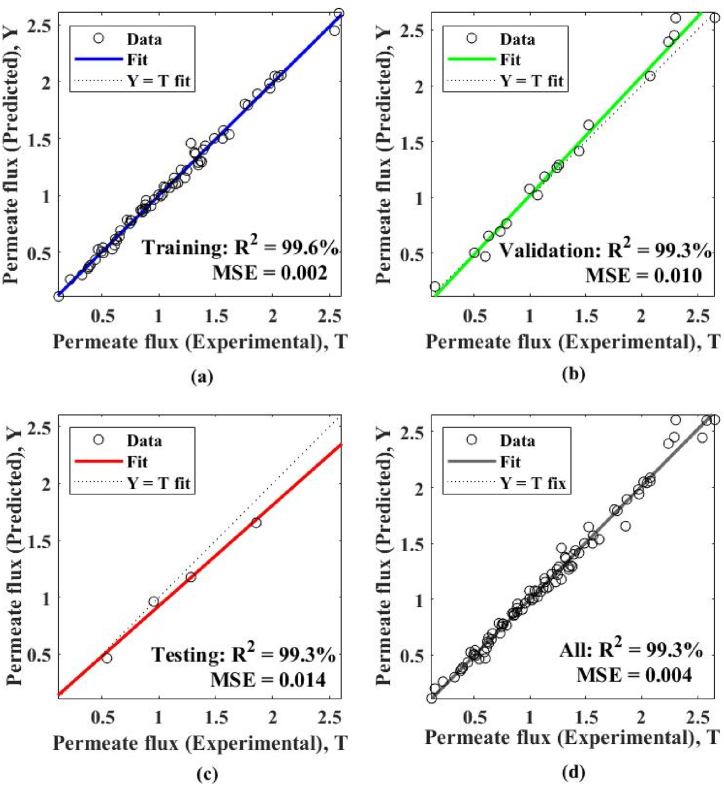
Scatter plots for the performance of plant of Model‒4 in all stages: (a) training, (b) validation, (c) testing, and (d) all.

This research discovered the best four models (IGWO-ANN Model‒1 to 4) after optimizing the proposed model (as shown in Table 4). The outputs of these models are superior to those of existing models (RSM, ANN, and GWO-ANN). In the best four models, IGWO-ANN Model‒4 achieves the best-of-best performance. As a result, This research has gone into in-depth about Model‒4 in this section. As shown in Fig. 4, simulation results reveal that Model‒4 has a quick convergence rate of performances in all stages (training, validation, and testing). Therefore, it may be able to reduce the modeling's execution time. Furthermore, a scatter plot (Fig. 5) demonstrated that Model‒4 produces productive outcomes for all stages. In Fig. 5, all stages {training (R^2^ = 99.6 %, MSE = 0.002), validation (R^2^ = 99.3 %, MSE = 0.010), testing (R^2^ = 99.3 %, MSE = 0.014), and all (R^2^ = 99.3 %, MSE = 0.004)} perform >99 % performances with minimum errors, indicating a better modeling approach. Thus, the simulation results suggest that the predicted permeate flux closely matches the experimental.

**Table 4:** The evaluation with current models [35,36]. *Employed the same experimental datasets for modeling as existing models; SA*** indicates the number of grey wolves, while the number of hidden layer neurons is shown by n**.

[A] Current Models:		Results {*P*_flux_ (L/h.m^2^)}	
References	Model Names	R^2^ (%)	Error
Gil et al., 2018 [35]	1. RSM Model	98.5	0.100
Gil et al., 2018 [35]	2. ANN Model	98.8	0.060
Mahadeva et al., 2022 [36]	3. GWO-ANN Model‒1	98.8	0.008
Mahadeva et al., 2022 [36]	4. GWO-ANN Model‒2	98.9	0.007
Mahadeva et al., 2022 [36]	5. GWO-ANN Model‒3	98.8	0.008
Mahadeva et al., 2022 [36]	6. GWO-ANN Model‒4	98.8	0.008

**[B] Proposed Models:**				
	n**	SA***		
1. IGWO-ANN Model‒1	9	15	98.8	0.007
2. IGWO-ANN Model‒2	13	15	98.8	0.008
3. IGWO-ANN Model‒3	3	9	98.8	0.007
4. IGWO-ANN Model‒4	**9**	**9**	**99.3**	**0.004**

**Fig. 6:**
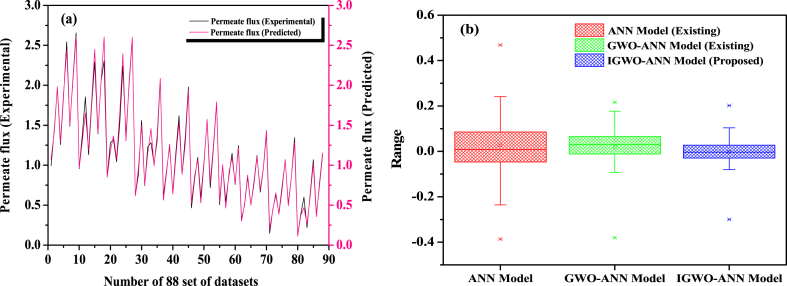
(a) Illustration of predicted *versus* experimental plant outcomes (permeate flux) of the proposed IGWO-ANN Model‒4. **(b)** Box plot for IGWO-ANN Model‒4.

For a better view, a comparison of the proposed models' experimental and predicted permeate flux values is shown in Fig. 6 (a). It shows that the experimental and predicted values match closely for Model‒4. As a result, this research concludes that the proposed technique predicts the best global solutions in search space and provides optimal weights and biases for ANN modeling.

### 3.3 Comparison with existing models

As demonstrated in Table 4, the updated and improved proposed models (IGWO-ANN Models 1 through 4) perform better than the existing ANN, GWO-ANN, and RSM models. The simulation results suggested they are highly effective timesaving and optimization tools. In addition, nine hidden layer neurons and search agents (n = SA = 9) are most suitable for model optimization. Furthermore, when compared to existing models, the proposed model had the lowest errors in the range of (−0.1 to 0.1), as shown in the box plot (Fig. 6 (b)), whereas the existing ANN model reported (between −0.3 and 0.3) and the GWO-ANN model reported errors (between −0.1 and 0.2). Therefore, it concluded that the proposed model performs better with minimum errors and can be optimized more than current models.

### 3.4 Limitations and future recommendations

During the simulation of this research, mainly noted three limitations.

(1) Dataset size and quality: Literature suggests that dataset size and quality play an important role in the modeling. Small and diverse datasets are not enough to accurately predict the performance. However, this research employed 88 sets of input-output experimental datasets (provided by Gill et al., 2018) and produced better results.
(2) Computational Complexity: A large number of modeling parameters (such as hidden layers, hidden layer nodes, number of grey wolves, and optimization function) make computational complexity. This research tried to minimize modeling parameters to increase computational speed and performance.
(3) Overfitting: During simulation, sometimes overfitting is recorded; however, a systematic and step-by-step approach has been eliminated.

Future recommendations.

(1) Larger and more diverse datasets: Future research should aim to gather larger datasets from various desalination plants under different operating conditions. This can help improve the model's robustness and generalizability.
(2) Hybrid models: Explore integrating other optimization techniques with ANN or hybrid models combining physical-based models and machine-learning approaches to enhance predictive accuracy and interpretability.
(3) Real-time implementation: Investigate ways to reduce the computational load, such as model simplification or more efficient algorithms, to facilitate real-time implementation and broader applicability.

By addressing these limitations and recommendations, future research can enhance the utility and accuracy of IGWO-ANN predictive models, contributing to more efficient and effective desalination plant operations.

## 4 Conclusion

Artificial intelligence technologies have played a key role in designing, optimizing, and managing industrial plant performance in recent decades. An Artificial Neural Network (ANN) model based on the Improvised Grey Wolf Optimizer (IGWO) is presented in this study to improve the performance of the RO desalination plant. In order to do this, four input variables (feed flow rate and salt content, evaporator and condenser intake temperatures) and one output variable (permeate flux) were considered. Furthermore, the models' accuracy has been evaluated using regression coefficients (R^2^) and mean squared error (MSE). The main conclusions of the present investigation are as follows: (a) Four models (IGWO-ANN-1, IGWO-ANN-2, IGWO-ANN-3, and IGWO-ANN-4) were suggested in this work to help forecast the plant's permeate flux precisely and accurately. (b) The Improvised GWO-ANN‒4 model shows a strong optimization ability, provides better outcomes with minimum errors, and outperforms the current models {ANN, Response Surface Methodology (RSM), and GWO-ANN}. (c) The improvised models (all four) require only (9–15) grey wolves to optimize the performance, whereas the existing model (GWO-ANN models) requires 15 grey wolves. Therefore, it reduces the model's complexity and the computational execution time. (d) In addition, the proposed models require only 3 to 13 hidden layer neurons (*n* = 3 to 13) in the modeling, whereas the exiting models (GWO-ANN) require larger hidden layer neurons (*n* = 15). (e) Finally, it is shown that there is a significant degree of similarities between the experimental and predicted permeate flux of the plants. The best thing about the proposed models is that they yield exact results, which may help scientists plan and control the operation of industrial plants. Lastly, this study's unique feature is the application of an IGWO algorithm to optimize the ANN model's parameters, which has been shown to increase ANN dependability.

## 5 Availability of data and materials

Data and materials will be made available on request.

## Funding

This work was supported by the 10.13039/501100004070Khalifa University, Abu Dhabi, 10.13039/100016565UAE The project (FSU-2022-030-Project Code: 8474000453) provided funding for this work.

## CRediT authorship contribution statement

**Rajesh Mahadeva:** Writing – review & editing, Writing – original draft, Visualization, Validation, Methodology, Data curation, Conceptualization. **Mahendra Kumar:** Validation, Supervision, Methodology, Data curation, Conceptualization. **Anjali Diwan:** Methodology, Data curation, Conceptualization. **Gaurav Manik:** Writing – review & editing, Supervision, Conceptualization. **Saurav Dixit:** Writing – review & editing, Methodology, Conceptualization. **Gobind Das:** Supervision, Data curation, Conceptualization. **Vinay Gupta:** Supervision, Conceptualization. **Anuj Sharma:** Writing – review & editing, Supervision, Methodology, Data curation, Conceptualization.

## Declaration of competing interest

The authors declare that they have no known competing financial interests or personal relationships that could have appeared to influence the work reported in this paper.
