# Supplementary material for: Improvised grey wolf optimizer assisted artificial neural network (IGWO-ANN) predictive models to accurately predict the permeate flux of desalination plants
Source: Heliyon. 2024 Jul 5;10(13):e34132. doi: 10.1016/j.heliyon.2024.e34132 (PMC11277383; doi:10.1016/j.heliyon.2024.e34132)
Supplement: Multimedia component 1 [file mmc1.pdf]

# Supplementary materials

Experimental dataset for IGWO-ANN modeling (Gil et al., 2018)

| Inputs | | | | Output |
|---|---|---|---|---|
| **S (g/L)** | **$T_{cond}$ (°C)** | **$T_{evap}$ (°C)** | **F (L/h)** | **$P_{flux}$ (L/(h·m$^2$ ))** |
| 35 | 20 | 60 | 400 | 0.995 |
| 35 | 20 | 70 | 400 | 1.44 |
| 35 | 20 | 80 | 400 | 1.973 |
| 35 | 20 | 60 | 500 | 1.257 |
| 35 | 20 | 70 | 500 | 1.867 |
| 35 | 20 | 80 | 500 | 2.545 |
| 35 | 20 | 60 | 600 | 1.487 |
| 35 | 20 | 70 | 600 | 2.077 |
| 35 | 20 | 80 | 600 | 2.656 |
| 35 | 25 | 60 | 400 | 0.954 |
| 35 | 25 | 70 | 400 | 1.378 |
| 35 | 25 | 80 | 400 | 1.856 |
| 35 | 25 | 60 | 500 | 1.13 |
| 35 | 25 | 70 | 500 | 1.756 |
| 35 | 25 | 80 | 500 | 2.293 |
| 35 | 25 | 60 | 600 | 1.391 |
| 35 | 25 | 70 | 600 | 2.048 |
| 35 | 25 | 80 | 600 | 2.306 |
| 35 | 30 | 60 | 400 | 0.854 |
| 35 | 30 | 70 | 400 | 1.281 |
| 35 | 30 | 80 | 400 | 1.32 |
| 35 | 30 | 60 | 500 | 1.043 |
| 35 | 30 | 70 | 500 | 1.525 |
| 35 | 30 | 80 | 500 | 2.241 |
| 35 | 30 | 60 | 600 | 1.365 |
| 35 | 30 | 70 | 600 | 2.019 |
| 35 | 30 | 80 | 600 | 2.583 |
| 60 | 20 | 60 | 400 | 0.618 |
| 60 | 20 | 70 | 400 | 0.888 |
| 60 | 20 | 80 | 400 | 1.56 |
| 60 | 20 | 60 | 500 | 0.745 |
| 60 | 20 | 70 | 500 | 1.23 |
| 60 | 20 | 80 | 500 | 1.281 |

| | | | | |
|---|---|---|---|---|
| 60 | 20 | 60 | 600 | 1.016 |
| 60 | 20 | 70 | 600 | 1.313 |
| 60 | 20 | 80 | 600 | 2.076 |
| 60 | 25 | 60 | 400 | 0.612 |
| 60 | 25 | 70 | 400 | 0.931 |
| 60 | 25 | 80 | 400 | 1.24 |
| 60 | 25 | 60 | 500 | 0.654 |
| 60 | 25 | 70 | 500 | 1.166 |
| 60 | 25 | 80 | 500 | 1.62 |
| 60 | 25 | 60 | 600 | 0.884 |
| 60 | 25 | 70 | 600 | 1.347 |
| 60 | 25 | 80 | 600 | 1.978 |
| 60 | 30 | 60 | 400 | 0.466 |
| 60 | 30 | 70 | 400 | 0.84 |
| 60 | 30 | 80 | 400 | 1.095 |
| 60 | 30 | 60 | 500 | 0.591 |
| 60 | 30 | 70 | 500 | 1.032 |
| 60 | 30 | 80 | 500 | 1.566 |
| 60 | 30 | 60 | 600 | 0.72 |
| 60 | 30 | 70 | 600 | 1.194 |
| 60 | 30 | 80 | 600 | 1.782 |
| 87.5 | 25 | 60 | 500 | 0.506 |
| 87.5 | 20 | 70 | 500 | 0.996 |
| 87.5 | 25 | 70 | 400 | 0.544 |
| 87.5 | 25 | 70 | 500 | 0.882 |
| 87.5 | 25 | 70 | 600 | 1.148 |
| 87.5 | 30 | 70 | 500 | 0.76 |
| 87.5 | 25 | 80 | 500 | 1.246 |
| 140 | 20 | 60 | 400 | 0.324 |
| 140 | 20 | 70 | 400 | 0.496 |
| 140 | 20 | 80 | 400 | 0.853 |
| 140 | 20 | 60 | 500 | 0.501 |
| 140 | 20 | 70 | 500 | 0.79 |
| 140 | 20 | 80 | 500 | 1.12 |
| 140 | 20 | 60 | 600 | 0.663 |
| 140 | 20 | 70 | 600 | 0.958 |
| 140 | 20 | 80 | 600 | 1.407 |
| 140 | 25 | 60 | 400 | 0.147 |

| | | | | |
|---|---|---|---|---|
| 140 | 25 | 70 | 400 | 0.441 |
| 140 | 25 | 80 | 400 | 0.628 |
| 140 | 25 | 60 | 500 | 0.396 |
| 140 | 25 | 70 | 500 | 0.732 |
| 140 | 25 | 80 | 500 | 1.06 |
| 140 | 25 | 60 | 600 | 0.511 |
| 140 | 25 | 70 | 600 | 0.861 |
| 140 | 25 | 80 | 600 | 1.345 |
| 140 | 30 | 60 | 400 | 0.118 |
| 140 | 30 | 70 | 400 | 0.385 |
| 140 | 30 | 80 | 400 | 0.6 |
| 140 | 30 | 60 | 500 | 0.22 |
| 140 | 30 | 70 | 500 | 0.63 |
| 140 | 30 | 80 | 500 | 1.068 |
| 140 | 30 | 60 | 600 | 0.376 |
| 140 | 30 | 70 | 600 | 0.753 |
| 140 | 30 | 80 | 600 | 1.135 |
